# Feasibility of community level interventions for pre-eclampsia: perspectives, knowledge and task-sharing from Nigeria, Mozambique, Pakistan and India

**DOI:** 10.1186/s12978-016-0245-6

**Published:** 2016-09-30

**Authors:** Mary V. Kinney, Jeffrey Michael Smith, Tanya Doherty, Jorge Hermida, Karen Daniels, José M. Belizán

**Affiliations:** 1 Save the Children, Cape Town, South Africa; 2 Jhpiego, Baltimore, MD USA; 3 Health Systems Research Unit, South African Medical Research Council, Cape Town, South Africa; 4 School of Public Health, University of the Western Cape, Bellville, South Africa; 5 Center for Human Services, University Research Company, Bethesda, USA; 6 Department of Mother & Child Health Research, Institute for Clinical Effectiveness and Health Policy (IECS), Buenos Aires, Argentina

## Abstract

Hypertensive disorders of pregnancy (HDP), particularly pre-eclampsia and eclampsia, remain one of the leading causes of maternal mortality and are contributory in many foetal/newborn deaths. This editorial discusses a supplement of seven papers which provide the results of the first round of the CLIP (Community Level Interventions for Pre-eclampsia) Feasibility Studies. These studies report a number of enablers and barriers in each setting, which have informed the implementation of a cluster–randomized trial (cRCT) aimed at reducing pre-eclampsia-related, and all-cause, maternal and perinatal mortality and major morbidity using community-based identification and treatment of pre-eclampsia in selected geographies of Nigeria, Mozambique, Pakistan and India. This supplement unpacks the diverse community perspectives on determinants of maternal health, variant health worker knowledge and routine management of HDP, and viability of task sharing for preeclampsia identification and management in select settings. These studies demonstrate the need for strategies to improve health worker knowledge and routine management of HDP and consideration of expanding the role of community health workers to reach the most remote women and families with health education and access to health services.

A healthy mother and baby are the desired outcomes of all antenatal and intrapartum care. Hypertensive disorders of pregnancy (HDP), particularly pre-eclampsia and eclampsia, remain one of the leading causes of maternal mortality and are contributory in many foetal/newborn deaths. As a result, an estimated 76,000 maternal and 500,000 foetal/newborn deaths occur each year with more than 10 million pregnancy complications [1]. The vast majority of deaths occur in low and- middle-income countries (LMICs), particularly in South Asia and Sub-Saharan Africa, due to deficiencies in early identification, triage, transport, and treatment, including provider competence, availability of basic supplies, and flawed care processes. The achievement of the new targets in the Sustainable Development Goals to end preventable maternal and newborn mortality will require universal access to improved delivery of evidence-based solutions for preventable conditions, such as HDP.

Improving quality of care for mothers and babies during pregnancy and childbirth has the potential to prevent 2 million deaths each year [2], yet to reach the most rural and vulnerable families, we need to increase demand, access and quality of care for the hardest to reach populations. While facility-based interventions are broadly recommended and called for, community based approaches have also demonstrated substantial impact on maternal and perinatal mortality outcomes through strategies such as participatory women’s groups, mobile technology, home visits and task sharing [3–7]. Despite the eagerness to generate evidence, and translate this into policy, implementation strategies require careful consideration as national circumstances vary [8–10].

Specific to HDP, research is now underway through the CLIP (Community Level Interventions for Pre-eclampsia) trials in hopes of reducing the burden of adverse maternal and perinatal outcomes related to pregnancy hypertension through community engagement, mobile technology and task-sharing to community health-care providers [11]. Using an individually powered, cluster–randomized trial (cRCT) design, the goal of the CLIP trials are to reduce pre-eclampsia-related, and all-cause, maternal and perinatal mortality and major morbidity by 20 % or more in intervention clusters using community-based identification and treatment of pre-eclampsia in selected geographies of Nigeria, Mozambique, Pakistan and India [12].

This editorial discusses a supplement of seven papers which provide the results of the first round of the CLIP Feasibility Studies [13–19]. These qualitative studies aimed to explore prevailing facilitators and barriers for the upcoming implementation of the CLIP cRCT by exploring community perspectives on determinants of maternal health, health worker knowledge, including community health workers (CHW), routine management or pregnancy and task sharing. These studies determined the feasibility of conducting the CLIP cRCT in selected sites using a similar mixed methods approach across the four study sites, tailored, however, to each individual cultural setting and health system context [20].

Understanding the perspectives of women and their communities offers important insights into the enablers and barriers to accessing health care. More context-specific studies and opportunities for women themselves to be engaged in defining quality of maternal and newborn care are needed. One paper in this supplement specifically focuses on community perspectives on the determinants of maternal health in Mozambique, including women’s perspectives, although literature from other settings is referenced in other papers [19]. In Mozambique, communities identified a broad range of factors that influence maternal health, including political, economic, socio-cultural and environmental factors [19]. Yet in other settings, community misconceptions about HPD result in the placement of blame on the woman and family and falsely implicate socio-cultural determinants, such as intimate partner violence [13, 19]. These findings underscore the need for consistent, contextualized community engagement about the actual risk factors and determinants of maternal health as well as the appropriate roles of heath care providers, including the CHW.

These studies further elucidate the issues of health worker knowledge and routine pregnancy management related to HDP. For CHWs to effectively educate communities about HDP and serve their communities in identifying and managing cases, the CHWs themselves need to have the knowledge about the etiology, symptoms and treatment options available. Across the different settings of the CLIP Feasibility studies, the knowledge of CHWs regarding pre-eclampsia and eclampsia spanned a broad range: Lady Health Workers (LHWs) in Pakistan and Community Health Extension Workers (CHEWs) in Nigeria were correctly able to identify cases [13, 14]; Accredited Social Health Activists (ASHAs) in India had some misconceptions about the etiology but were able to identify cases [16]; and CHWs in Mozambique were able to identify pregnancy complications but not specifically preeclampsia and eclampsia [17]. Other health care providers, particularly doctors, demonstrated sufficient knowledge to diagnose HDP and provide routine management.

Magnesium sulfate – the World Health Organization (WHO) recommended medication for the treatment and prevention of seizures related to severe pre-eclampsia [21] - is inexpensive and appropriate for women in low-resource settings; however implementation challenges remain due to lack of availability, fear of adverse effects, confusion regarding routes of administration, and dosing uncertainty [22]. This supplement provides further evidence to this affect. These studies show that despite the presence of national guidelines, there is need for more broad dissemination and promotion to clarify the scope of practice for each health care provider and where required, such as in Nigeria, to remove regulatory restrictions preventing certain categories of workers from managing pre-eclampsia in community settings [13, 14, 16, 17]. In all four settings of the CLIP studies, the authors recommend that management of preeclampsia be included in regular trainings of health care providers, depending on their role in that setting, with protocols provided at all health facilities.

These studies further examined the feasibility of task-sharing aspects of care for women with HDP [15, 18], in response to the global shortage of healthcare workers, particularly in the most remote settings, and how that shortage impacts adequate provision of maternal health care. There is evidence that task-sharing with other cadres, such as CHWs, can play a substantial role in maternal health – in certain contexts and under certain circumstances [7, 23]. While these findings are encouraging, further work needs to be done to sustain and scale-up task-sharing programmes. Task-sharing requires immense dedication, coordination, and leadership as well as defined roles with standardized and assessed competency levels and supportive supervision [6, 22, 24, 25]. Task-sharing also requires supportive policy and regulations regarding specific tasks, as well as strategies to protect lay health workers from liability [22].

The two papers in this supplement on task sharing provide further important evidence on known strategies [15, 18] including that team work and team building is vital. While the CHEWS and LHWs may report similar knowledge about preeclampsia, the different team structures in place will ultimately determine task-sharing prospects. The paper from Pakistan concludes that the LHW programme has the potential to expand the role of the LHWs in recognition of the trust that both users (the women and their families) and providers have in the established, functioning system [15]; whereas the paper from Nigeria finds that barriers in training, community engagement and policies need to be addressed before selected maternal health tasks could be appropriately shared with CHEWs [18]. Furthermore, experience with the current informal task-sharing with CHEWs, a result of health worker shortage in Nigeria, has raised concerns about their capacity to undertake additional tasks that would take them beyond their current mandate [18]. Task sharing requires trust among the team, buy in from all stakeholders, clear delineated roles and supportive supervision.

In several of these countries, the CHWs are multi-purpose, delivering as many as 23 activities and services including nutrition, newborn care, health promotion and prevention and curative care for children [26]. With the current renewed interest in community-based delivery platforms as a mechanism to achieve universal access to health care, certain parameters are important to monitor during the course of the CLIP trials in order to enhance the interpretation and understanding of the trial findings. As more tasks are shared with community providers, the density or ratio of CHWs to families is a critical factor influencing impact. This has been shown in relation to community-based treatment of severe-acute malnutrition [27]. Monitoring the ratio of CHWs to families in these trial sites will therefore be important to understand the influence of this parameter on intervention effectiveness. Other important process indicators which are likely to mediate the intervention effect would include community trust and demand for services, reliability of the supply chain, frequency of supervision, including clinical supervision and staff attrition. The CLIP trials will provide important information to inform the global consultations on health policy and system support to optimize CHW programmes [28].

The authors of the CLIP Feasibility Studies report a number of enablers and barriers in each setting, which have informed the implementation of the currently underway CLIP cRCT. This supplement adds to the knowledge base of HDP by revealing the diverse community perspectives on determinants of maternal health, variant health worker knowledge and routine management on HDP, and viability of task sharing for preeclampsia identification and management in select settings. These studies demonstrate the need for strategies to improve health worker knowledge and routine management of HDP and consideration of expanding the role of CHWs to reach the most remote women and families with health education and access to health services.

We hope the readers of Reproductive Health find this supplement to be a rich resource for further understanding how best to improve the quality of maternal and perinatal health care in these settings and beyond.
